# Evaluating the impact of topological protein features on the negative examples selection

**DOI:** 10.1186/s12859-018-2385-x

**Published:** 2018-11-20

**Authors:** Paolo Boldi, Marco Frasca, Dario Malchiodi

**Affiliations:** Department of Computer Science, Università degli Studi di Milano, Via Comelico 39, Milano, 20135 Italy

**Keywords:** Negative example selection, Protein function prediction, Biological networks, Protein features

## Abstract

**Background:**

Supervised machine learning methods when applied to the problem of automated protein-function prediction (*AFP*) require the availability of both positive examples (i.e., proteins which are known to possess a given protein function) and negative examples (corresponding to proteins not associated with that function). Unfortunately, publicly available proteome and genome data sources such as the Gene Ontology rarely store the functions *not* possessed by a protein. Thus the *negative selection*, consisting in identifying informative negative examples, is currently a central and challenging problem in *AFP*. Several heuristics have been proposed through the years to solve this problem; nevertheless, despite their effectiveness, to the best of our knowledge no previous existing work studied which protein features are more relevant to this task, that is, which protein features help more in discriminating reliable and unreliable negatives.

**Results:**

The present work analyses the impact of several features on the selection of negative proteins for the Gene Ontology (GO) terms. The analysis is network-based: it exploits the fact that proteins can be naturally structured in a network, considering the pairwise relationships coming from several sources of data, such as protein-protein and genetic interactions. Overall, the proposed protein features, including local and global graph centrality measures and protein multifunctionality, can be *term-aware* (i.e., depending on the GO term) and *term-unaware* (i.e., invariant across the GO terms). We validated the informativeness of each feature utilizing a temporal holdout in three different experiments on yeast, mouse and human proteomes: (i) feature selection to detect which protein features are more helpful for the negative selection; (ii) protein function prediction to verify whether the features considered are also useful to predict GO terms; (iii) negative selection by applying two different negative selection algorithms on proteins represented through the proposed features.

**Conclusions:**

Term-aware features (with some exceptions) resulted more informative for problem (i), together with node *betweenness*, which is the most relevant among term-unaware features. The node *positive neighborhood* instead is the most predictive feature for the *AFP* problem, while experiment (iii) showed that the proposed features allow negative selection algorithms to select effectively negative instances in the temporal holdout setting, with better results when nonlinear combinations of features are also exploited.

**Electronic supplementary material:**

The online version of this article (10.1186/s12859-018-2385-x) contains supplementary material, which is available to authorized users.

## Background

The publicly available databases devoted to record protein functions (for instance, the Functional Catalogue [1] and the Gene Ontology [2]) typically contain entries associating a protein with the biological functions the protein is known to possess. On the other hand, these repositories rarely consider *not possessed* functions. Thus, if a protein is not associated with a function, this could be simply due to a lack of information. Indeed, in such cases it is not possible to exclude that future studies could in principle associate that protein with that function.

Among the available protein function taxonomies, this work considers the Gene Ontology (GO), a hierarchy composed of three branches, biological process (BP), molecular function (MF), and cellular component (CC), each structured as a direct acyclic graph [2]. The functions described in this ontology (referred to as *GO terms*) are often (positively) annotated solely to a small number of proteins. Therefore, remaining proteins either do *not possess* the function, or correspond to not yet discovered positive annotations.

This observation leads to a central and critical issue in the problem of automated protein-function prediction (*AFP*), consisting in discovering novel associations of proteins with biological functions through computational methodologies. Indeed, the automated prediction process is typically based on *supervised*/*semi-supervised* machine learning techniques, requiring both positive and negative associations of proteins with functions (technically referred to as *positive* and *negative examples*, respectively) in order to infer accurate predictors. In this context, selecting the negative examples is a central issue for *AFP* [3–5]. The methods proposed in the literature to tackle the negative selection problem typically rely on bagging (bootstrap aggregating) techniques, based on the repeated inference of binary classifiers discriminating positive examples from reliable subsets of non-positive examples. These subsets are obtained through random subsampling on non-positive examples [6], either being guided by specific positive-negative similarity measures [7–9], or simply subsampling the items under the assumption that the probability to get a false positive be sufficiently small [10]. In addition, some heuristics have been proposed specifically for the *AFP* context, negatively associating a term with all proteins positive for sibling and/or ancestral GO terms [11], or computing the empirical conditional probability of a term given the annotations for other terms in the three GO branches, considering all nodes [4] or only the hierarchy leaves [12].

To our knowledge no researches have tried to investigate the possible relations between suitable ‘protein features’ and the fact that a protein can be considered as a reliable negative example. That is, before applying any algorithm to learn negative examples, it is of paramount importance studying which ‘protein representation’ is more informative for the problem itself. In this context, most information sources about the relationships between proteins are naturally represented through protein networks, where each node represents a protein and an edge the relationship between two proteins [13]; additionally, most approaches proposed for *AFP* are network-based [14–20]. Thus, the purpose here is twofold: extracting meaningful protein features from protein networks, and assessing their ability to improve the identification of good negative examples.

By extending the study presented in [21], this paper proposes a set of 14 features, ranging from protein multifunctional properties, to local and global graph centrality measures, including weighted degree, betweenness, and clustering centrality. Such features have been divided in the *term-aware* and *term-unaware* subsets, referring respectively to features varying with the GO term under study and to features independent of GO terms. With a dedicated experiment, the significance of each feature for selecting negatives has been assessed by adopting a state-of-the-art feature selection algorithm, along with a temporal holdout setting, necessary to determine the category of proteins not reliable as negative examples (that is, those that received a novel annotation in the holdout period). Through the paper this category is denoted by *C*_*np*_ (the **c**ategory of **n**egative proteins that become **p**ositive). As further validation, in another experiment the proposed features have been provided as input to two procedures for learning negatives, evaluating their ability in detecting proteins not in *C*_*np*_. In the above mentioned analyses we also tested 3 probabilistic features computed by *3Prop*, a state-of-the-art method to extract features from biological networks; the results of *3Prop* have already been tested against the *AFP* problem [22], but their use within the negative selection has not been investigated yet. Finally, another experiment has been set up to predict the GO protein functions, to get more insight about the information encoded in the 14 proposed features. Overall, our paper extends the research done in [21] by adding 8 novel features, by performing feature selection on temporal holdout data, by applying linear and nonlinear state-of-the-art methods to learn negative examples, and by constructing extended and updated datasets for three organisms (yeast, mouse, and human).

Our studies showed that the set of features informative for identifying negative examples depends on both the organism and the GO branch considered. As a trend common to different settings, term-aware features tend to be selected more frequently, especially *Positive neighborhood* and *Mean of positive neighborhood*. Term-unaware features, however, play an important role, with some differences among organisms: *Neighborhood mean* and *Weighted clustering coefficient* are more frequently selected in yeast, whereas *Betweenness* is largely more informative in mouse and human. The most predictive feature for the *AFP* problem is *Positive neighborhood*: indeed, when representing proteins by eliminating just this feature, the highest decrease in performance is observed. When providing the proposed protein representation as input to negative selection algorithms, our 14 features allow linear methods to achieve the lowest number of false negatives (that is, proteins in *C*_*np*_ classified as reliable negatives), which on the contrary increases when adding *3Prop* features to the representation, or when representing proteins just using *3Prop*. Finally, when using nonlinear methods to learn negatives, the number of false negatives largely decreases, and it is nearly the same when adopting the proposed features and *3Prop*; this phenomenon is likely due to novel information coming from nonlinear interactions of the *3Prop* features that linear methods are not able to exploit.

The paper is organized as follows: a first section describes the adopted methodology, including data description, the proposed features, and the setting of the different experiments carried out. The second section reports the obtained results and the related discussion, while some concluding remarks close the paper.

## Methods

This section aims at describing the data sources leveraged in order to construct protein networks and protein functional annotations, the protein features extracted, and their experimental validation. Three different experiments have been performed to validate the adopted features:

- assessing feature relevance,
- predicting protein functions,
- selecting reliable negative proteins.

Each of the above mentioned steps is described in detail in the following sections.

### Data

The input networks have been retrieved from the STRING database, version 10.0 [23], for the following organisms: *S.cerevisiae* (yeast), *Mus musculus* (mouse) and *Homo sapiens* (human). The STRING network already merges several sources of data, including protein homology relationships from different species, thus resulting in a highly informative network. Connections in such a network are endowed with a “combined score” that represents how reliable that relation should be considered; as suggested by STRING curators, connections with a combined score lower than 700 (combined scores range from 1 to 999) were filtered out. The network topological characteristics are reported in Table 1. All input networks have one large and some smaller connected components. The total number of nodes does not include nodes that became isolated after edge thresholding. Networks have been normalized as described in the next section.

**Table 1:** Description of data networks

Organism	Nodes	Average degree	Components	Component size	Diameter	Weighted diameter
Yeast	5586	38.4740	41	5483, 2–7	12	3.0481
Mouse	13921	59.9990	190	13417, 2–10	13	3.1857
Human	15154	47.5572	89	14951, 2–10	11	3.1552

Column Components denotes the number of connected components in the network, whereas Component size denotes the corresponding number of nodes. Diameter is the number of edges on the longest path between two nodes, without considering edge weights

Functional annotations for STRING proteins have been downloaded from the Gene Ontology, by considering two different temporal releases: the UniProt GOA releases 69 (9 May 2017) and 40 (25 November 2014) for yeast, releases 155 (6 June 2017) and 125 (25 November 2014) for mouse, and releases 168 (9 May 2017) and 139 (25 November 2014) for human. The two releases form a ‘temporal holdout’: the older release is used for the training phase, and the later release allows to evaluate the quality of predictions. In both releases, solely experimentally validated annotations have been considered. The relevance assessment of node/protein features to detect reliable negatives was focused on proteins which received at least a new annotation during the temporal holdout period (for a given GO term); we denote by *C*_*np*_ this category of proteins. Then we selected the GO terms with at least 20 proteins in *C*_*np*_, obtaining the terms summarized in Table 2. The proposed features were also tested in terms of their capability in predicting the protein functions, by selecting GO terms with 20–200 annotations in the later release, in order to have a minimum of information to train a classifier, and to exclude terms with a large number of annotations, because they are too generic [13, 24, 25]. The total number of obtained GO terms is shown in Table 3.

**Table 2:** Number of GO terms in the three GO branches for which |*C*_*np*_|≥20. |*C*_*np*_| denotes the cardinality of *C*_*np*_ (i.e., the number of negative proteins that become positive in the temporal period)

Organism	CC	MF	BP
Yeast	5	9	29
Mouse	62	75	512
Human	71	105	363

**Table 3:** Number GO terms with 20–200 annotated proteins in the more recent release

Organism	CC	MF	BP
Yeast	9	18	41
Mouse	18	32	178
Human	41	64	153

### Preliminaries

Protein networks are represented as an undirected graph *G*〈*V*,***W***〉, with *V*={1,…,*n*} denoting the set of nodes/proteins and ***W*** being a *n*×*n* matrix whose entries *W*_*ij*_∈[0,1] encode some notion of intra-protein functional similarity (with *W*_*ij*_=0 when the corresponding nodes are not connected). The matrix ***W*** is obtained from the STRING connections $\hat {\boldsymbol {W}}$ after the following normalization, which preserves the connection symmetry:

$$\boldsymbol{W} = {\boldsymbol{D}}^{-1/2} \hat{\boldsymbol{W}} \boldsymbol{D}^{-1/2} $$

where ***D*** is a diagonal matrix with non-null elements $d_{ii} = {\sum \nolimits }_{j} \hat W_{ij}$. The temporal holdout validation scheme relies on two additional matrices $\boldsymbol {Y},\overline {\boldsymbol {Y}} \in \{0,1\}^{n\times m}$ containing the annotations of proteins to *m* GO terms {1,…*m*}: each matrix refers to a different temporal release of the ontology (assuming ***Y*** as the older one). If we denote the *r*-th column and the *i*-th row of a matrix ***X*** by ***X***_.*r*_ and ***X***_*i*._ respectively, then ***Y***_.*k*_ and $\overline {\boldsymbol {Y}}_{.k}$ describe the annotations for the GO term *k* to the proteins in *V* at the beginning and at the end of the houldout period. Moreover, *N*_*i*_:={*j*∈*V*|*W*_*ij*_≠0} denotes the neighborhood of node *i*∈*V*, and for a given GO term *k*, $N_{i}^{+} := \{j \in N_{i} | Y_{jk}=1\}$ denotes its positive neighborhood, that is the subset of the neighborhood composed only of nodes positively annotated for *k* (Here, as in many of the following notations, the index *k* of the GO term is left implicit).

We recall that fixed a term *k*, *C*_*np*_⊆*V* is the set of proteins that received a new annotation in the holdout period, that is, $C_{np} = \{i\in V | Y_{ik}=0 \wedge \overline Y_{ik}=1 \}$.

As mentioned in the previous section, the main aim of this paper is extracting features from nodes in *G* which effectively discriminate proteins belonging to *C*_*np*_ from proteins negatively annotated in both releases, as shown in the next section.

### Extracting proteins features

The protein features studied in this work are selected in order to consider on the one hand information about the network topology, including both local and global ‘standard’ node centrality measures, on the other hand information about protein annotations. The resulting set of protein features is shown in Table 4.

**Table 4:** The considered features for node *i*∈*V* and GO term *k*

Symbol	Name	Definition
*f*1	Neighborhood mean	$\frac {1}{|N_{i}|}\sum \limits _{j\in N_{i}} W_{ij}$
*f*2	Neighborhood variance	$\frac {1}{|N_{i}|-1}\sum \limits _{j\in N_{i}} (W_{ij}-f1(i))^{2}$
*f*3	Weighted degree	$\sum \limits _{j\in N_{i}} W_{ij}$
*f*4	Weighted clustering coefficient	$ {\sum \limits _{j,j' \in N_{i}, j' \in N_{j}} \frac {W_{ij}+W_{ij'}+W_{jj'}}3}\left / {\sum \limits _{j,j' \in N_{i}} \frac {W_{ij}+W_{ij'}}2} \right.$
*f*5	Number of annotations	$\sum \limits _{h=1}^{m} Y_{ih}$
*f*6	Closeness centrality [33]	$\frac 1{{\sum \nolimits }_{j \in C_{i}}d_{ij}}$
*f*7	Lin’s index [34]	$\frac {\left |C_{i}\right |^{2}}{{\sum \nolimits }_{j \in C_{i}}d_{ij}}$
*f*8	Harmonic centrality [32]	$\sum \limits _{j \in C_{i}}\frac 1{d_{ij}}$
*f*9	Betweenness [35, 36]	$\sum \limits _{s,t \in C_{i}, s \neq i, t \neq i, s \neq t} \frac {\sigma _{st}(i)}{\sigma _{st}}$
*f*10	Positive neighborhood	$\sum \limits _{j\in N_{i}^{+}} W_{ij}$
*f*11	Mean of positive neighborhood	$\frac {1}{|N_{i}^{+}|}\sum \limits _{j\in N_{i}^{+}} W_{ij}$
*f*12	Positive closeness centrality	$\frac 1{{\sum \nolimits }_{j \in C_{i}^{+}}d_{ij}}$
*f*13	Positive Lin’s index	$\frac {\left |C_{i}^{+}\right |^{2}}{{\sum \nolimits }_{j \in C_{i}^{+}}d_{ij}}$
*f*14	Positive harmonic centrality	$\sum \limits _{j \in C_{i}^{+}}\frac 1{d_{ij}}$
*f*15	1-step Random Walk	***P***_*i*._ ***y***
*f*16	2-step Random Walk	$\boldsymbol {P}^{2}_{i.} \,\boldsymbol {y}$
*f*17	3-step Random Walk	$\boldsymbol {P}^{3}_{i.} \,\boldsymbol {y}$

*C*_*i*_ denotes the connected component of *i*, $C_{i}^{+}$ the positive nodes in *C*_*i*_, *d*_*st*_ the shortest-path distance from *s* to *t* (using ***W*** as weight matrix), *σ*_*st*_ the number of shortest paths from *s* to *t*, and *σ*_*st*_(*u*) the number of such paths that include *u* as internal node. ***P*** and ***y*** are defined in (1) and (2)

A first group of features depends only on the structure of the network *G*: some of them are purely *local*, in the sense that they exploit a limited local neighborhood around the protein of interest (*f*1– *f*4); other features are more *global* in nature (*f*6– *f*9), and correspond broadly to some of the most common parameter-free centrality measures in network analysis. A second group of features, besides using the network structure, takes also into consideration the annotations (*f*5) or refers to the term-aware variant of some of the features of the first two groups (*f*10– *f*14). The considered features are summarized in Table 4 and described here below. *f*1*Neighborhood mean*: mean of connection weights in the protein neighborhood. *f*2*Neighborhood variance*: variance of connection weights in the protein neighborhood. *f*3*Weighted degree*: sum of connection weights in the protein neighborhood. *f*4*Weighted clustering coefficient*: weighted proportion of triplets centered in the protein of interest that turn out to be closed (i.e. triangles). *f*5*Number of annotations*: number of GO terms for which the protein is annotated in the older release. *f*6*Closeness centrality*: reciprocal of the sum of shortest-path distances from the protein to all the other proteins in the same connected component. *f*7*Lin’s index*: an adjusted version of closeness, obtained multiplying it by the square of the size of the component. *f*8*Harmonic centrality*: sum of the reciprocal of all the shortest-path distances from the protein to all the other reachable proteins. *f*9*Betweenness*: sum of the fractions of shortest paths that pass through the given protein. *f*10*Positive neighborhood*: sum of connection weights in the protein positive neighborhood. *f*11*Mean of positive neighborhood*: mean of connection weights in the protein positive neighborhood. *f*12*Positive closeness centrality:* reciprocal of the sum of shortest-path distances from the protein to all the positive proteins in the same connected component. *f*13*Positive Lin’s index*: an adjusted version of positive closeness, obtained multiplying it by the square of the number of positive proteins in the same connected component. *f*14*Positive harmonic centrality*: sum of the reciprocal of all the shortest-path distances from the protein to all the positive reachable proteins.

The first two features refer to the first moments of the distribution of connection weights in the neighborhood of a node. The third feature provides information about the node connectivity, and moreover has been suggested in the literature as a proxy for gene multifunctionality [26, 27]. Jointly considering the first and third feature conveys information about the number of connections, one of the main measures for the connectivity of nodes in graphs along with the weighted degree [28].

Measure *f*4 is the weighted-aware version of the local clustering coefficient [29] of the node under consideration: for each triplet centered in the node, we compute its average weight (the average weight of the two or three edges involved). The ratio between the total weight of closed triples and total weight of all triples gives the local clustering coefficient; this quantity coincides with the standard (local) clustering coefficient when all the weights coincide. It is a variant of the weighted version proposed in [30] that takes into full account all the three weights appearing in the closed triples.

Feature *f*5 is related to the ability of a protein to play different roles: in its computation, the current GO term has been excluded in order to not introduce bias.

Features *f*6– *f*8 are among the most classical geometric centrality measures. As many authors observe [31, 32], closeness centrality *f*6 [33] (essentially, up to a constant, the reciprocal of the average distance between the node under consideration and the other nodes in its component) provides biased results in presence of disconnected components with largely different sizes; Lin’s index *f*7 [34] and harmonic centrality *f*8 [32] both try to mitigate this big-in-Japan effect in different ways (one by explicitly taking the size of the connected component into account, and the other by looking implicitly at the distance from all nodes, using harmonic average instead of arithmetic average—where infinite distances give a null contribution). Another quite classical centrality measure is betweenness *f*8, originally defined by Anthonisse for edges [35] and then adapted by Freeman to nodes [36]; this index measures robustness rather than centrality (it is related to the probability that shortest-path routing fails when the node is deleted).

Feature *f*10 instead exploits both the number of positive neighbours and the corresponding weight magnitudes, and it plays the role of a *guilt-by-association* score [16]. Together with *f*10, feature *f*11 describes the number of connections toward positive nodes. Overall, features *f*1– *f*9 are *term-unaware*, in the sense that they do not need the annotation vector ***Y***_.*k*_ to be computed (for a given term *k*). A special case is represented by the feature *f*5, which uses an information not directly related to the annotations for the current GO term, but encompassing GO terms; hence, we did not include it in the group of term-aware features. Conversely, features *f*10 and *f*11 are the term-aware versions of *f*1 and *f*3, respectively, and similarly *f*12– *f*14 are the term-aware equivalent of *f*6– *f*8.

In order to have comparable ranges, features have been normalized so as to sum up to one across proteins, that is ${\sum \nolimits }_{i=1}^{n}fk(i) = 1$, for each *k*∈{1,2,…14}.

It is worth pointing out that the centrality measures considered here do not cover all the indices examined in the literature [32], and in particular they do not include any spectral index: measures such as PageRank or Katz’s index were avoided in order to exclude the proliferation of parameters whose tuning would increase the chance of overfitting. Other spectral indices (such as Seeley’s index) do not apply to disconnected networks. For similar reasons, the consideration of scale-aware measures [37] was left as future work.

To further enrich our analysis, the state-of-the-art *3Prop* features have also been considered: originally proposed to extract features from biological networks, this algorithm describes a protein *i*∈*V* with three features $p_{i}^{j}$, *j*=1,2,3, each representing the probability that a random walk (respectively of length 1, 2 and 3) which starts from a positive (annotated) node ends in *i* [22]. Namely, fixed a GO term *k*, recalling the previously introduced diagonal matrix ***D***, and setting

1 $$ \boldsymbol{P}=\boldsymbol{D}^{-1}\boldsymbol{W},\\ \boldsymbol{y}=(y_{1}, \ldots, y_{n}),  $$

with components

2 $$ y_{i} = \left\{\begin{array}{ll} \frac{1}{\sum_{h} Y_{hk}} &\ \text{if} {i} \text{is annotated for} {k} \\ 0 &\ \text{otherwise,} \end{array}\right.  $$

it holds that $p_{i}^{j} = \boldsymbol {P}^{j}_{i.} \boldsymbol {y}$ for *j*∈{1,2,3} (where of course ***P***^2^=***PP***, and ***P***^3^=***P***^2^***P***). Features $p_{i}^{1}$, $p_{i}^{2}$ and $p_{i}^{3}$ (*f*15– *f*17 in Table 4) are thereby included in the group of term-aware features.

### Assessing feature relevance

To evaluate the efficacy of node features *f*1– *f*17 described in Table 4 in detecting reliable negative proteins (i.e., those neither annotated in the first release nor in the second one), a binary classification problem was established. In this problem, proteins are represented through the extracted features, and their label is provided by the class *C*_*np*_ for GO terms in Table 2. The aim is selecting the features which mostly improve the classification performance. As classifier we adopted the *CART* algorithm [38], combined with the *Sequential floating forward Search* (SFFS) method [39] to determine the optimal subset of features. We employed the SFFS algorithm to capture the combined effect of multiple features; due to the potentially large number of add/remove steps until convergence, SFFS requires an efficient classifier, such as *CART*, which in addition is able to exploit feature interactions. To prevent selection bias and overfitting, data were partitioned into three non-overlapping subsets (following the setting proposed in [25]). On each subset a triple-loop of 3-fold cross-validation (CV) has been executed, using training data to select the classifier model (through the inner CV loop). Such model has been used on the corresponding test fold to validate the current subset of features.

In order to deal with the scarcity of positive instances, the *F*_1_ measure was selected as performance criterion to be maximized both in the inner and the outer CV loops. Finally, we ranked features through the proportion of times they have been selected in all the experiments over the three data subsets.

### Predicting protein functions

The proposed features have also been tested in classifying node/proteins to the Gene Ontology terms, to assess their capability in capturing network structures useful for the automated protein-function prediction. For each term previously described and summarized in Table 3, a binary classification problem was set up, with proteins represented in turn through features *f*1– *f*14, *f*15– *f*17, and *f*1– *f*17. In order not to have any bias toward a specific classifier, two state-of-the-art methods were used to solve the binary classification problems where instances were represented through feature vectors: *linear support vector machines* (SVM) with class weights [40] and *Random Forests* (RF) [41]. The performance has been evaluated using a 3-fold outer loop CV, and a 3-fold inner loop CV to select the parameters *C* and *mtry*, respectively for SVM and RF models. To counterbalance the large presence of negative examples and to avoid learning trivial models, the class weights of the SVM for term *k* have been set to 1 and $\frac {n-{\sum \nolimits }_{i=1}^{n} Y_{ik}}{{\sum \nolimits }_{i=1}^{n} Y_{ik}}$ for the negative and the positive class, respectively, as suggested in [42]. The *F*_1_ measure has been adopted both to select the model and to measure the classification performance (averaged across folds), since this measure is more informative when positive instances are rare. Furthermore, results are also reported in terms of *Precision* (proportion of annotated proteins among those classified as positive) and *Recall* (proportion of annotated proteins that were positively classified).

### Selecting reliable negative proteins

**Fig. 1:**
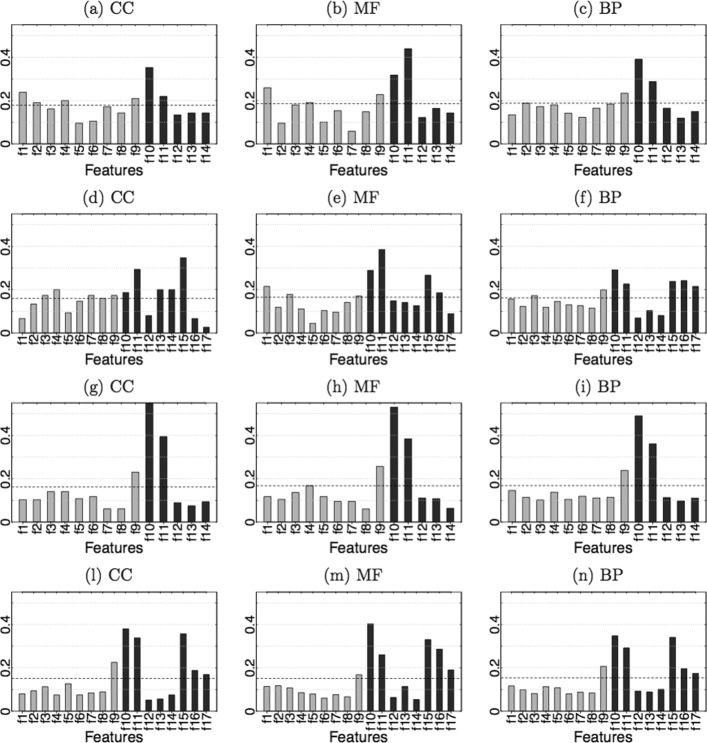
Proportion of times features are selected by the *SFFS* algorithm on *yeast* (first two rows) and *human* (last two rows) data. Grey and black bars are for term-unaware and term-aware protein features. The black horizontal dashed line corresponds to the mean value of the bars. For each organism, the two rows refer to the use of features *f*1– *f*14 and *f*1– *f*17, respectively. **a**, **d**, **g**, **l** correspond to CC terms, **b**, **e**, **h**, **m** to MF terms, and **c**, **f**, **i**, **n** to BP terms

In order to study further the subsets of features that help detecting reliable negatives, the features described in Table 4 were supplied as input to negative selection algorithms, to investigate the relevance of the following different combinations of features:

- *f*15– *f*17,
- *f*1– *f*14 - top *q*,
- *f*1– *f*14 - mean,
- *f*1– *f*14,
- *f*1– *f*17 - top *q*,
- *f*1– *f*17 - mean,
- *f*1– *f*17,

where ‘top *q*’ denotes the selection of the top *q*=5 features in the corresponding ranking in Fig. 1 and Additional file 1, and ‘mean’ denotes the selection of the features having a frequence larger than the mean frequency value (black dashed horizontal line in the figures). The choice *q*=5 derives from the observation of frequency distributions: in some cases just three features are above the mean, while in other ones the distribution has a lower variance with seven features overcoming the mean frequency value. The value of *q* has been tuned as a compromise between these two different conditions.

The selection of reliable negatives was performed through protein ranking, both exploiting the decision function values for SVMs and leveraging the probability to belong to a given class in RFs. The negative proteins selected as reliable are those bottom-ranked by the models. Following the temporal holdout setting, the models were trained on the older annotation release, by fixing a budget of negatives to be selected, subsequently computing the number of false negatives averaged across terms (those reported in Table 3) using the annotations in the newer release. The budget was set as the *x%* of the total number of proteins, with *x*∈{1,5,10,15,20,25,30}.

## Results and discussion

### Assessing feature relevance

To better evaluate the informativeness of graph centrality measures in classifying proteins in *C*_*np*_, feature selection has been performed by representing proteins both using solely centrality features *f*1– *f*14 and using all features *f*1– *f*17. Figure 1 depicts the obtained frequencies for *yeast* and *human* organism (the results for *mouse* are shown in Additional file 1). In most experiments *Positive neighborhood* is the most informative feature, in both settings adopted. Also *Mean of positive neighborhood* and *1-step Random Walk* are frequently selected, being the top feature respectively on MF branch for yeast data (Fig. 1b, 1e), and on CC branch for yeast and mouse data (Fig. 1d, Additional file 1(d)). Term-aware features (black bars) tend to be predominant over those that are term-unaware, with an exception represented by the *Betweenness centrality*, often more informative than some term-aware centralities, and nearly the top selected one in mouse (Additional file 1(d-f)). Overall, results for human and mouse show more similar trends than yeast: this fact is probably due to more similar topological structures of the corresponding protein networks (see Table 1), and to the fact that human and mouse are phylogenetically closer to each other than yeast.

Notably, betweenness appears to be much more informative than other geometric centrality measures (e.g., closeness); this outcome is in line with the general observation that betweenness is scarcely correlated with most of the remaining centrality indices, and it may be associated to the fact that the considered networks have a relatively small diameter.

*Number of annotations* seems to carry a significant signal on mouse, mainly for CC and BP branches (Additional file 1(d, f)), whereas non negligible frequence enhancing are seen for *Weighted clustering coefficient* (yeast – CC), and for *Neighborhood mean* (yeast – MF).

The two settings *f*1– *f*14 and *f*1– *f*17 provide to some extent analogous results, with some differences that however seem not to be related to an underlying physical topology: for instance, *Neighborhood mean* has a significantly higher frequency when discarding *3Prop* features on yeast CC data (Fig. 1a, d), but on yeast BP (Fig. 1c, f) and mouse MF (Additional file 1(b, e)) the opposite happens.

In summary, among term-unaware centralities only the *Betweenness centrality* is significantly enhanced in the majority of experiments, thus helping in discriminating reliable and unreliable negative examples. On the other hand, *Positive closeness, Positive Lin’s* and *Positive harmonic* centralities are likely to be useless for this task. Finally, the relevance of *3Prop* features in detecting reliable negatives is decreasing with the number of steps of the random walk.

### Protein function prediction

**Fig. 2:**
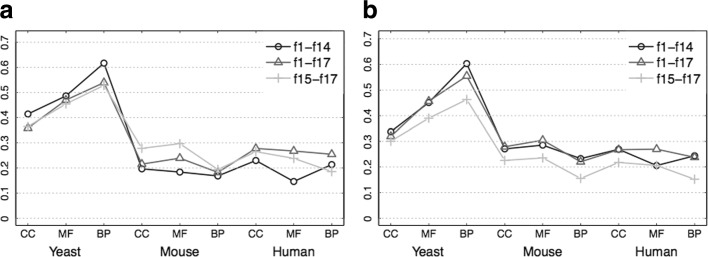
Performance in terms on *F*_1_ measure averaged across GO branches for linear SVM (**a**) and RF (**b**) classifiers

The classification performances in terms of *F*_1_ measure are summarized in Fig. 2, whereas *Precision* and *Recall* results are shown in Additional file 2(a-b) and (c-d), respectively. Interestingly, concerning yeast data, centrality measures allow both classifiers to achieve the best results, even better than those obtained when the *3Prop* features are added to the protein representation (*f*1– *f*17). Such results are confirmed also in terms of *Recall*. On mouse and human data, *f*1– *f*14 representation is still more informative than *f*1– *f*17 when employing RFs, performing similarly to *3Prop* representation. Conversely, SVMs achieve the best results when using all features, and this is likely due to the ability of RFs in capturing the combined effect of features, thus making some of them redundant; on the other hand, SVMs need a more complex protein representation to achieve nearly the same results. SVMs also tend to have a higher *Recall*, while RFs are more precise. This is probably due to the adoption of cost-sensitive SVM learning, which, by attributing a larger misclassification weight to positive instances, tends to increase both the number of true and false positives.

**Fig. 3:**
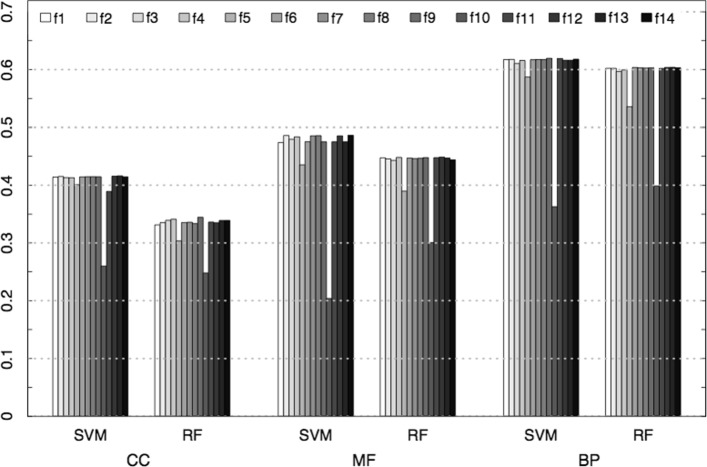
Average *F*_1_ across GO branch terms on yeast data when removing the corresponding feature

In addition, to give an insight about the impact of each feature on the automated protein-function prediction, each classification experiment was repeated removing in turn one feature and using all the remaining features to represent proteins. The results in terms of *F*_1_ are summarized in Fig. 3: *3Prop* features have been excluded from this experiment because their effectiveness in predicting GO functions has already been assessed in [22]. Due to its complexity, we ran this procedure solely on yeast data. Analogous results based on precision and recall are shown in Additional files 3 and 4, respectively.

Clearly, the most informative measure is *Positive neighborhood* (*f*10), whose removal causes the largest decrease in *F*_1_ values. The removal of *Number of annotations* (*f*5) leads to a significant decay for both classifiers, whereas when singularly eliminating the other features just negligible differences can be observed. These results clearly show that some features are redundant for this task, and the application of feature selection methodologies may lead to better results than those depicted in Fig. 2.

### Selecting Negatives

**Fig. 4:**
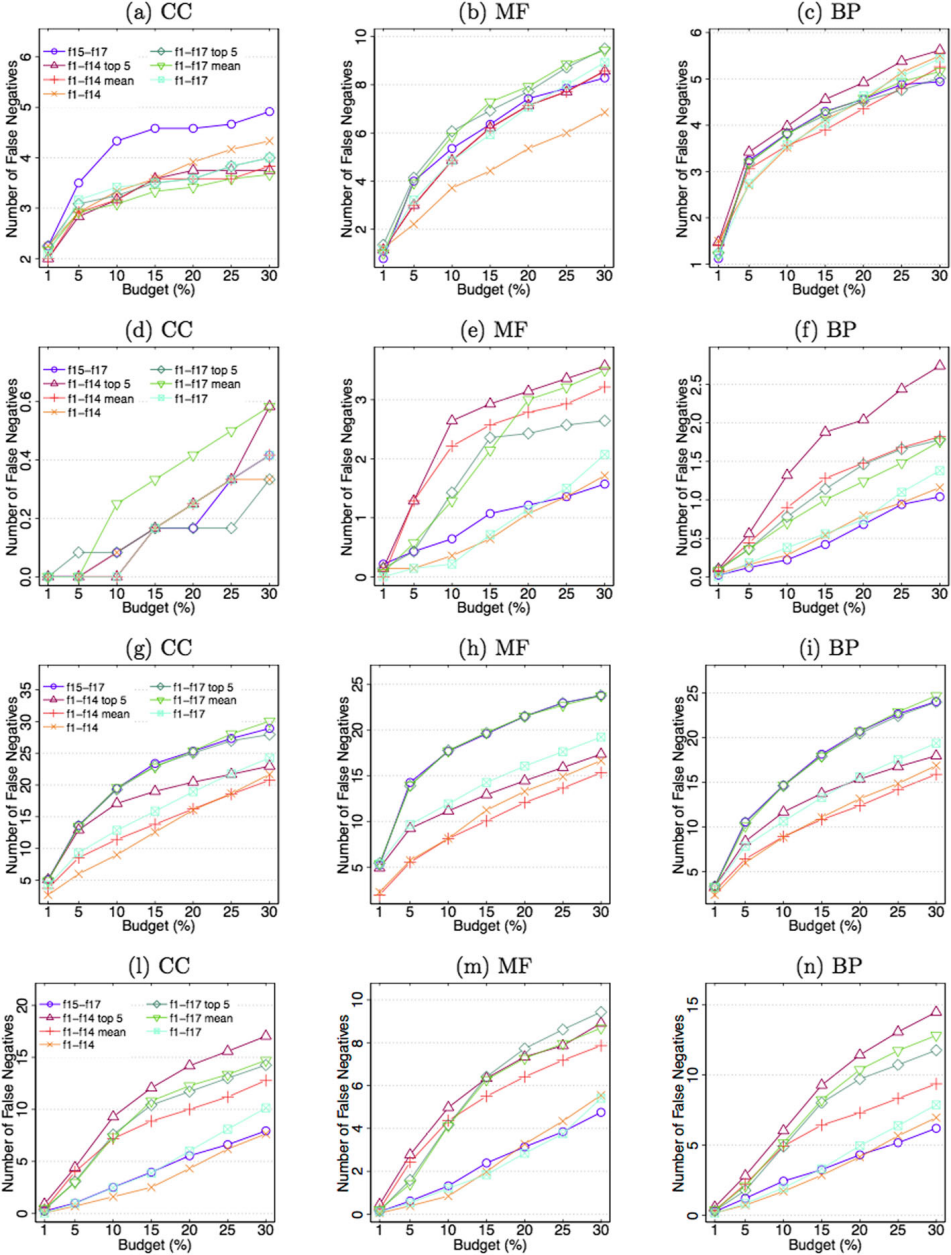
Number of false negatives averaged across GO terms. Results in the first two rows are obtained on *yeast* data, whereas the last two rows refer to *human* data. First (resp. second) and third (resp. fourth) rows show the results of the SVM (resp. RF) selection algorithm. **a**, **d**, **g** correspond to CC terms, **b**, **e**, **h** to MF terms, and **c**, **f**, **i** to BP terms

Figure 4 reports the results of the negative selection for yeast and human organisms, whereas the corresponding results for mouse are shown in Additional file 5. A first interesting insight is that when adopting the RF selection method, the number of false negatives significantly decreases compared to the results obtained by the linear SVM selection, suggesting the need of using algorithms able to exploit interactions among features. Considering specific experiments, according to the results of SVM on CC terms and yeast data, the subset of features *f*1– *f*17* mean* is slightly the most informative, whereas *f*15– *f*17 (*3Prop*) achieve the (largely) worst performance. On BP terms most feature sets perform similarly, whereas on MF data the *f*1– *f*14 set has the top performance. On human data, again *f*1– *f*14 and *f*1– *f*14*mean* achieve the top performance, with close results, while on mouse data the combination including all features (*f*1– *f*17) is the top performing one.

Different behaviors are observed when RF model is adopted as negative selection procedure: in all the experiments, *f*1– *f*14 and *f*1– *f*17 feature sets are the top performing ones, likely due to the fact that eliminating even the features with low absolute frequencies wastes some useful combined effects that the RF model, for its nonlinear nature, is able to exploit. Indeed, the *top 5* and *mean* feature sets have been selected on the basis of the absolute individual frequencies reported in Fig. 1 and Additional file 1, computed to provide a general trend of feature informativeness; nevertheless, for specific tasks also coupled frequencies, or more in general feature set frequencies, could help in selecting the optimal combination of features. Another interesting behaviour is related to the *3Prop* features: along with the above mentioned set of features, it represents the top performing set, as opposed to SVM results. Thus these three features, appropriately combined, may also provide information similar to that encoded in the features *f*1– *f*17. On the other side, this requires more complex selection algorithms, thus features *f*1– *f*14 are preferable when, for complexity reasons, simpler models must be adopted.

In summary, features *f*1– *f*14 seem to be more informative for selection algorithms not able to capture nonlinear combined effects among feature subsets, whereas they perform similarly to the *3Prop* feature set when selection methods with higher classification capabilities are adopted. Nevertheless, by excluding features *f*12– *f*14, since they are rarely selected by the feature selection algorithm, the computation of the remaining features can nicely scale when input size increases, since features *f*1– *f*9 can be computed offline, being not term-specific, and features *f*10 and *f*11 can be computed efficiently. Conversely, the *3Prop* features need to simulate 6 random walks on the whole network, which also should be row-normalized, affecting thereby scalability.

## Conclusions

Seventeen protein features in biological networks have been studied in this work to assess their ‘usefulness’ for selecting relevant negatives in the *AFP* context. State-of-the-art graph centrality measures, GO term-aware measures, and protein multifunctionality have been considered. Term-aware features resulted more informative for selecting reliable negative proteins through a state-of-the-art feature selection method in a temporal holdout setting, where the validation is carried out on the proteins that received novel annotations in the temporal holdout period. Among the remaining features, the node (protein) *betweenness* showed an interesting pattern, in particular on mouse data, where it is close to being the most relevant feature. The protein *positive neighborhood* instead is the most predictive feature for the *AFP* problem (that is, when the task to be predicted is the GO term itself). Finally, by supplying the proposed features as input to linear and nonlinear negative selection algorithms, we discovered that there is little or no redundancy among the features when their linear combination is adopted, whereas their nonlinear interaction also provides novel discriminative abilities to negative selection algorithms.

Overall, apart for those mentioned above, a clear and regular trend did not arise, thus suggesting further analyses under different settings and/or adding (discarding) some features as future investigations.

## Additional files

Additional file 1 **Figure S1.** Proportion of times each feature is selected by the *SFFS* algorithm on *mouse* data and CC (a-d), MF (b-e) and BP (c-f) terms. Same notations as in Fig. 1. (PNG 79 kb)

Additional file 2 **Figure S2.** Performance in terms on *Precision* (a-b) and *Recall* (c-d) measures averaged across GO branch terms when proteins are represented through *f*1– *f*14, *3Prop* (*f*15– *f*17), and *f*1– *f*17 features. Left and right columns correspond to SVM and RF results, respectively. (PNG 130 kb)

Additional file 3 **Figure S3.** Evaluation of the impact of features *f*1– *f*14 on the classification performance. Bars correspond to the *Precision* results averaged cross GO branch terms on yeast data when removing the related feature. (PNG 47 kb)

Additional file 4 **Figure S4.** Evaluation of the impact of features *f*1– *f*14 on the classification performance. Bars correspond to the *Recall* results averaged cross GO branch terms on yeast data when removing the related feature. (PNG 54 kb)

Additional file 5 **Figure S5.** Number of false negative averaged across GO terms on the *mouse* data. First (resp. second) row shows the results of the SVM (resp. RF) selection algorithm. (PNG 203 kb)
