# Correction to: Evaluating the impact of topological protein features on the negative examples selection

**DOI:** 10.1186/s12859-018-2545-z

**Published:** 2018-12-17

**Authors:** Paolo Boldi, Marco Frasca, Dario Malchiodi

**Affiliations:** Department of Computer Science, Università degli Studi di Milano, Via Comelico 39, 20135 Milano, Italy

**Correction to: BMC Bioinformatics 2018, 19(Suppl 14):417**

10.1186/s12859-018-2385-x

After publication of the original article [1], it was noticed that the dagger symbol indicating equal contribution wasn’t added next to the names of all authors. The author list in this correction article has been modified to represent this correctly.
