# Supplementary figures and images for: Evaluating the impact of topological protein features on the negative examples selection
Source: BMC Bioinformatics. 2018 Nov 20;19(Suppl 14):417. doi: 10.1186/s12859-018-2385-x (PMC6245585; doi:10.1186/s12859-018-2385-x)

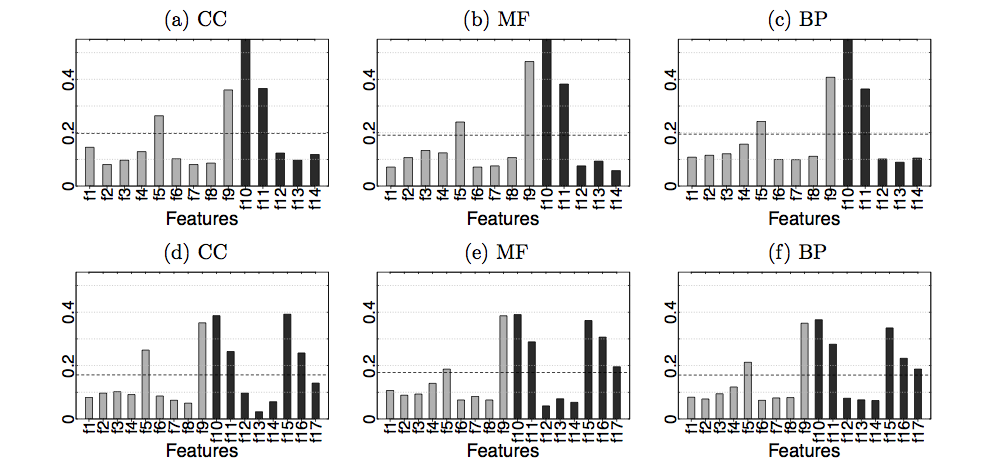

Supplement: Supplementary file 1 — Figure S1. Proportion of times each feature is selected by the SFFS algorithm on mouse data and CC (a-d), MF (b-e) and BP (c-f) terms. Same notations as in Fig. 1. (PNG 79 kb) [file 12859_2018_2385_MOESM1_ESM.png]

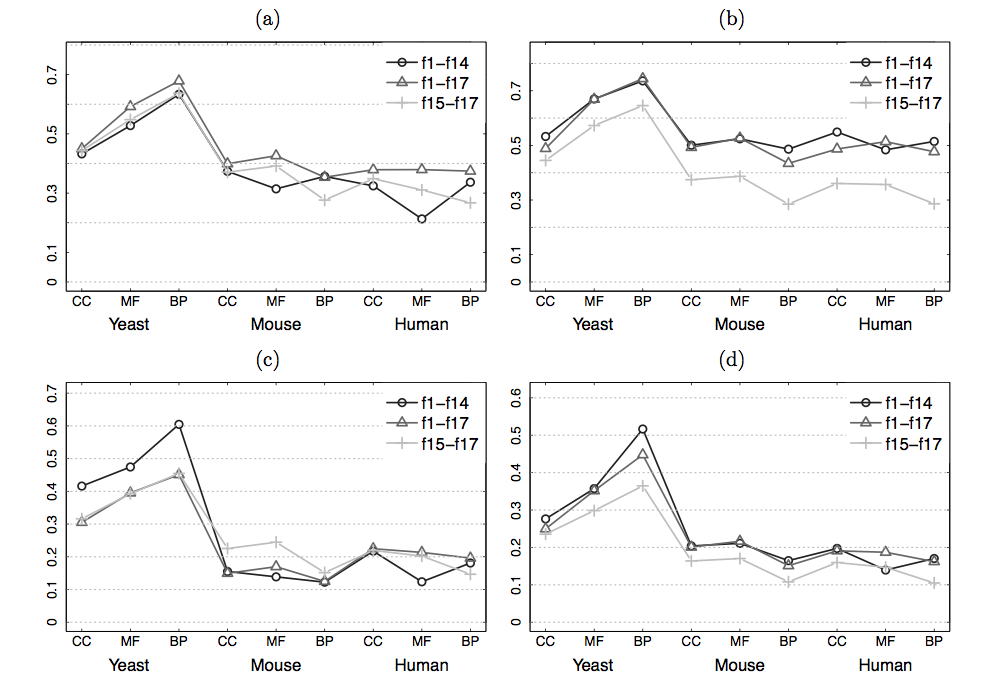

Supplement: Supplementary file 2 — Figure S2. Performance in terms on Precision (a-b) and Recall (c-d) measures averaged across GO branch terms when proteins are represented through f1– f14, 3Prop (f15– f17), and f1– f17 features. Left and right columns correspond to SVM and RF results, respectively. (PNG 130 kb) [file 12859_2018_2385_MOESM2_ESM.png]

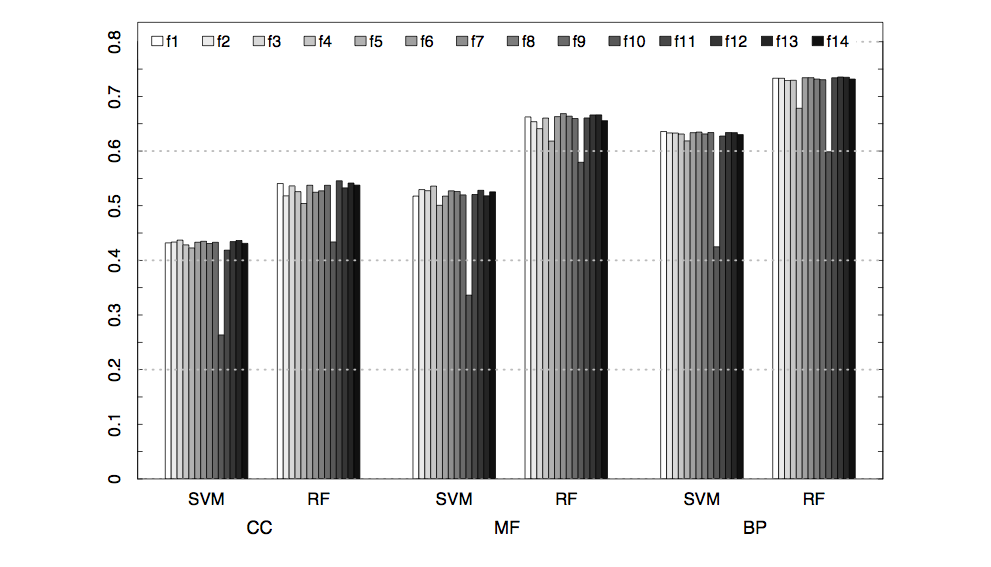

Supplement: Supplementary file 3 — Figure S3. Evaluation of the impact of features f1– f14 on the classification performance. Bars correspond to the Precision results averaged cross GO branch terms on yeast data when removing the related feature. (PNG 47 kb) [file 12859_2018_2385_MOESM3_ESM.png]

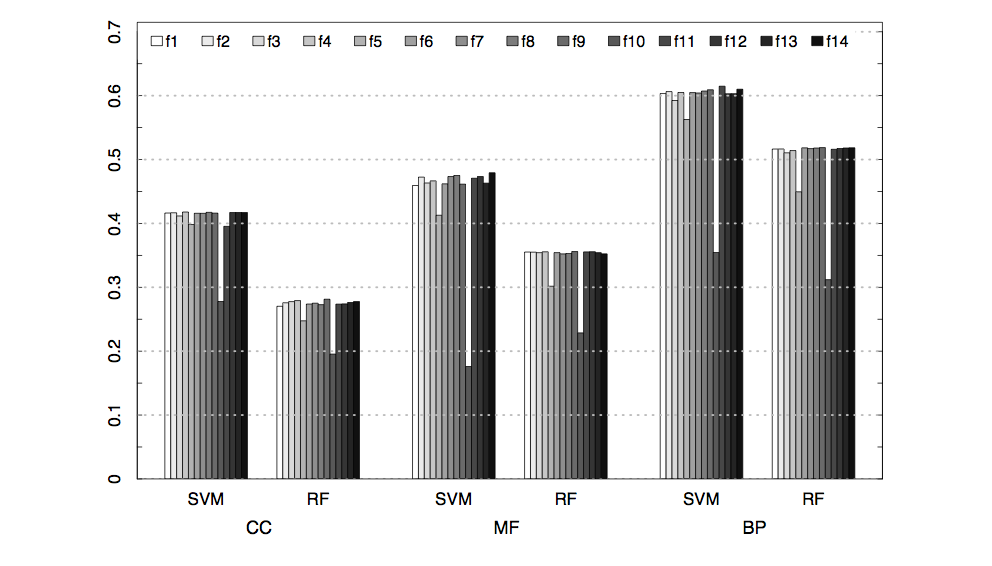

Supplement: Supplementary file 4 — Figure S4. Evaluation of the impact of features f1– f14 on the classification performance. Bars correspond to the Recall results averaged cross GO branch terms on yeast data when removing the related feature. (PNG 54 kb) [file 12859_2018_2385_MOESM4_ESM.png]

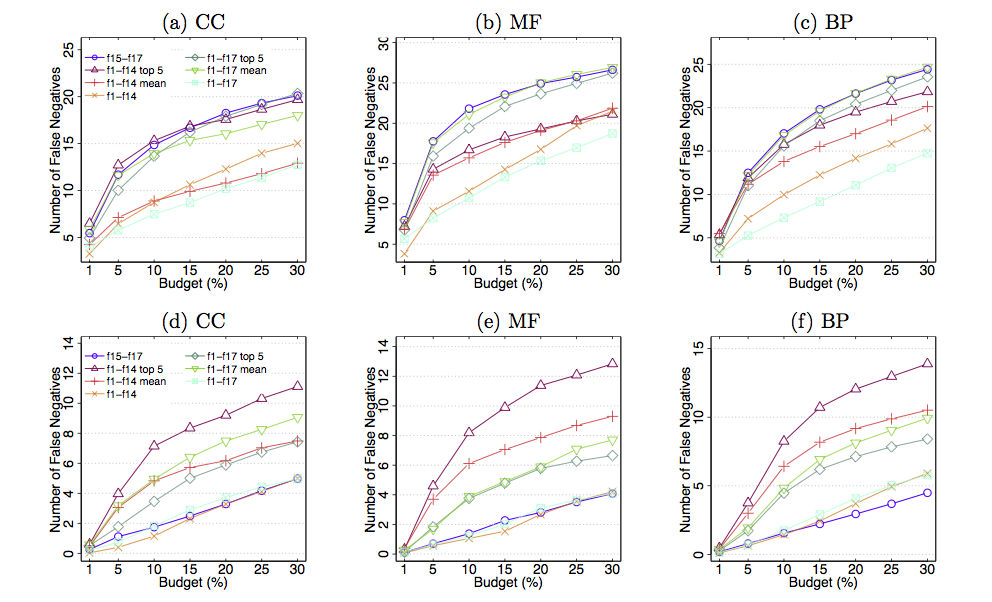

Supplement: Supplementary file 5 — Figure S5. Number of false negative averaged across GO terms on the mouse data. First (resp. second) row shows the results of the SVM (resp. RF) selection algorithm. (PNG 203 kb) [file 12859_2018_2385_MOESM5_ESM.png]
